# Social determinants of health as risk and protective factors for health care access among sexual and gender minority parents

**DOI:** 10.21203/rs.3.rs-7661476/v1

**Published:** 2025-10-24

**Authors:** Adary Zhang, Stephanie A. Leonard, Micah E. Lubensky, Annesa Flentje, Mitchell R. Lunn, Catherine Benedict, Diana M. Tordoff, Juno Obedin-Maliver

**Affiliations:** Stanford University School of Medicine; Stanford University School of Medicine; Stanford University School of Medicine; Stanford University School of Medicine; Stanford University School of Medicine; Stanford University School of Medicine; Stanford University School of Medicine; Stanford University School of Medicine

## Abstract

Sexual and gender minority (SGM) people are increasingly becoming parents. To examine the relationship between social determinants of health (SDOH) and health care access among SGM parents, we used 2018–2019 prospective cohort data from The PRIDE Study. We compared health care access between 555 SGM parents and 555 age-matched SGM non-parents. We then used modified Poisson regression to assess the association between SDOH at baseline and health care access at one-year follow-up among SGM parents. We found that SGM parents and SGM non-parents reported differences in SGM identity disclosure to health care providers and health care utilization. SGM parents were less likely than SGM non-parents to disclose SGM identity to health care providers (p < 0.001) and reported more health care avoidance (p = 0.021). Among SGM parents, greater SGM identity concealment (aRR 1.13, 95% CI 1.05–1.22) and increased social isolation (aRR 1.06, 95% CI 1.01–1.10) predicted increased health care avoidance attributed to fear of disrespect or mistreatment. Increased social isolation (aRR 1.05, 95% CI 1.01–1.09) also predicted increased all-cause delayed health care access. Among SGM parents, these proxy measures of interpersonal-level and community-level SDOH suggested risk and protective factors influencing health care access.

## Introduction

Sexual and gender minority (SGM) people constitute a diverse and increasingly visible population in the United States (US) and around the world.^1^ In the US, SGM people are increasingly becoming parents, with 2019 estimates indicating that at least 30% of US SGM adults are already parents and at least three million US SGM millennials are considering expanding their families.^2^

As parents, SGM people encounter numerous challenges across legal, social, and health domains regarding their parental rights and reproductive health outcomes.^3,4^ SGM parents often face barriers when seeking health care for their children, including challenges related to SGM identity disclosure, discrimination, and lack of competency with SGM-centered care in health care settings.^5,6^ Prior research has found that compared to SGM non-parents, SGM parents demonstrate differential mental health outcomes and health behaviors.^7,8^ There remains a knowledge gap regarding health-seeking behaviors and health care access among SGM parents. SGM parents likely face unique barriers, especially when considering the intersection of parenthood and other social determinants of health (*e.g.*, age, disability status).^7,9^ Social determinants of health have a large influence on health equity and health outcomes, and they include the wide range of non-medical individual, interpersonal, community, and structural factors that impact the conditions in which people work, live, play, and age.^10^ Minority stress and other social determinants of health often intertwine to synergistically mediate health inequities related to multiple minority identities.^11^

In recent years, a broadening evidence base has begun to describe how SGM people contend with numerous structural barriers to accessing health care, including stigma, discrimination, and bias at multiple levels of the health care system, with compounded challenges for those with multiple minority identities, such as SGM parents.^12^ An emerging body of research has also highlighted the strengths and resiliencies present within SGM populations.^13,14^ Research on supportive communities^15^ and community-centered interventions^16^ underscores the potential of leveraging various social determinants of health as protective factors to improve health outcomes and mitigate health inequities among SGM populations. SGM parents may benefit from protective effects of interpersonal and community support systems which may influence their health outcomes and health care utilization.^8^

Given the knowledge gap regarding health-seeking behaviors and health care access among SGM parents, this study sought to determine whether health care utilization patterns differ between SGM parents and SGM non-parents (*i.e.*, SGM people who are not parents). We also sought to identify social determinants of health associated with delayed health care access and health care avoidance attributed to fear of disrespect or mistreatment among SGM parents.

## Methods

### Theoretical framework.

We drew upon the Sexual & Gender Minority Health Disparities Research Framework,^17^ which is an SGM-specific adaptation of the National Institute on Minority Health and Health Disparities Research Framework.^18^ We applied this framework to examine social determinants of health among SGM parents. This framework conceptualizes health experiences, inequities, and resiliencies as dependent on a complex interplay between individual health characteristics, health behaviors, and social determinants of health. For this study, we examined proxy measures of social determinants of health across broadening levels of influence (*i.e.*, individual, interpersonal, and community) to assess their impact on health care access for SGM parents.

### Data source and study population.

We used 2018–2019 data from The Population Research in Identity and Disparities for Equality (PRIDE) Study, a national, US-based, online, prospective longitudinal cohort study of SGM adult health that combines a novel digital research platform with community-engaged recruitment and retention strategies.^19,20^ Our primary aim was to assess social determinants of health as predictors of health care utilization at one year follow-up. Therefore, our analysis included participants who responded to both the 2018 (hereafter referred to as baseline) and 2019 (hereafter referred to as follow-up) annual questionnaires and who answered questions about their parenthood status at baseline.

### Measures.

We defined parenthood status as whether participants responded “Yes” to the question “Are you a parent?” at baseline. Demographic characteristics were assessed at baseline and included age in years, ethnoracial identity, gender identity, sexual orientation, sex assigned at birth, annual household income in US dollars, marital status, relationship status, and self-reported disability status. For ethnoracial identity, gender identity, and sexual orientation, participants could select more than one response option. We created six mutually exclusive gender groups using participant responses to questions about gender identity and sex assigned at birth: cisgender men, cisgender women, gender diverse people who were assigned female at birth (AFAB), gender diverse people who were assigned male at birth (AMAB), transgender men, and transgender women.

We assessed social determinants of health at baseline as exposure variables as described above. As our proxy measure for individual-level social determinants of health, we included insurance status and type, which we combined into a single variable with the following categories: uninsured, private insurance, public insurance, VA/TRICARE, and other insurance. As our proxy measures for interpersonal-level social determinants of health, we included a binary indicator of whether participants reported currently having a primary care provider (PCP) and two continuous measures of SGM identity disclosure to and concealment from health care providers. We used items from a modified version of the Nebraska Outness Scale.^21^ The SGM identity disclosure items queried: “What percent of the people in this group do you think are aware of your gender identity (meaning they are aware of your gender or gender expression)?” and “What percent of the people in this group do you think are aware of your sexual orientation (meaning they are aware of whether you consider yourself straight, gay, etc.)?” Response options were in increments of 10% and ranged from 0% to 100%. The SGM identity concealment items queried: “How often do you avoid talking about topics related to or otherwise indicating your gender or gender identity (*e.g.*, not correcting people when they use a name or pronoun that is not accurate for you)?” and “How often do you avoid talking about topics related to or otherwise indicating your sexual orientation (*e.g.*, not talking about your significant other, changing your mannerisms) when interacting with health care providers?” Response options ranged from 0 (Never) to 10 (Always). As our proxy measures for community-level social determinants of health, we included two continuous measures of emotional support and social isolation. Both measures used the Patient-Reported Outcomes Measurement Information System (PROMIS) 4-item scales.^22^ We calculated T-scores from each participant’s raw score such that a T-score of 50 represented the US population mean with a standard deviation of 10, with higher T-scores indicating more of the measured construct (*e.g.*, higher emotional support, higher social isolation).

We considered two outcome variables assessed at follow-up: all-cause delayed health care access and health care avoidance attributed to fear of disrespect or mistreatment. We defined all-cause delayed health care access as whether participants responded “Yes” at follow-up to the question “In the past 12 months, were you delayed in getting medical care, tests, or treatments that you or a health care provider believed necessary?” We defined health care avoidance attributed to fear of disrespect or mistreatment as whether participants responded “Yes” at follow-up to the question “Was there a time in the past 12 months when you needed to see a health care provider but did not because you thought you would be disrespected or mistreated?”

## Statistical analysis.

We used descriptive statistics to evaluate baseline demographic characteristics and social determinants of health among SGM parents compared to SGM non-parents. We also compared SGM parents to SGM non-parents based on self-reported health care utilization at follow-up. Age is a notable confounder in health outcomes between SGM parents (median age 45 years) and SGM non-parents (median age 28 years)^7^ because older people are more likely to become parents, and thus SGM non-parents are always going to be significantly younger. Therefore, to control for confounding by age and focus on other demographic characteristics among SGM parents and SGM non-parents, we created a sample of age-matched SGM non-parents using propensity score matching to control for confounding by age. We estimated propensity scores using logit models and a 1:1 nearest neighbor matching algorithm without replacement to match on age only via the *MatchIt* package in R.^23^

We then restricted our sample to the cohort of SGM parents. We used regression models to explore the relationship between social determinants of health at baseline and health care access at one-year follow-up. We fit multivariable, modified Poisson regression models with robust standard errors to estimate relative risk (RR) and 95% confidence intervals. The outcomes of interest for all models were binary indicators of all-cause delayed health care access and health care avoidance attributed to fear of disrespect or mistreatment at follow-up among SGM parents. We selected confounders based on causal diagrams (Appendix A). We adjusted all models for age group, gender group, annual household income, and self-reported disability status. We did not adjust for other demographic variables, as overadjustment may mask the impact of demographic characteristics that affect access to health care. For example, though adjusting for annual household income statistically unbiases the relationship between social determinants of health and health care access, this adjustment may unintentionally mask the role of income as a proxy for social class and its attendant sociocultural and discriminatory effects on health care access.^24^ We used complete-case analysis given minimal missingness in our variables of interest.

All analyses were conducted using R statistical software (version 4.4.2).^25^ This study received ethics approval from the University of California, San Francisco; Stanford University Research Compliance Office; and WCG Institutional Review Boards. All research was performed in accordance with relevant guideline and regulations. Informed consent was obtained from all participants.

## Results

### Descriptive statistics.

There were 3,546 participants (555 SGM parents and 2,991 SGM non-parents) who met the inclusion criteria (Appendix B). After matching, our final sample included 555 SGM parents (hereafter “parents” in this section) and 555 age-matched SGM non-parents (hereafter “non-parents” in this section).

Baseline characteristics and social determinants of health are shown in Table 1. After age-matching, participants in both groups had a mean age of 44 years (Q1-Q3 37–56). The majority of both groups self-identified as white (94.2% among parents and 92.6% among non-parents), including those who selected multiple ethnoracial identities (6.8% and 6.4%). The ethnoracial identities of both groups were similar except that parents were more likely than non-parents to report Hispanic or Latinx identities (4.7% vs. 8.1%, p = 0.03). Parents and non-parents differed by gender (p < 0.001): parents were most commonly cisgender women (42.9%) and non-parents were most commonly cisgender men (47.6%). Annual household income, disability status, having a PCP, PROMIS emotional support and social isolation measures, and scores for identity concealment from health care providers were similar for parents and non-parents. However, parents had lower scores than non-parents (7.10 vs. 7.89, p < 0.001) for identity disclosure to health care providers (*i.e.*, parents were less likely than non-parents to feel that their health care providers were aware of their SGM identity).

Health care utilization at follow-up among parents and age-matched non-parents is shown in Table 2. Parents were more likely than non-parents to report health care avoidance in the past 12 months attributed to fear of disrespect or mistreatment (13.8% vs. 9.0%, p = 0.02). Parents and non-parents reported similar likelihoods of delaying (20.1% vs. 18.0%) or not receiving (13.1% vs. 10.9%) necessary health care in the past 12 months. Parents and non-parents also reported similar likelihoods when citing particular reasons for delaying necessary health care, and the most common of these reasons were lack of insurance coverage, being unable to be scheduled by a provider in a timely fashion, and being unable to afford care. There was overlap in these health care utilization metrics: 73% of participants who reported not receiving necessary health care also reported delayed health care access, and 32% of participants who reported delayed health care access also reported health care avoidance attributed to fear of disrespect or mistreatment.

### Delayed health care access among SGM parents.

As shown in Table 3, among parents (n = 488), higher scores for identity concealment from health care providers (RR 1.09, 95% CI 1.03–1.16) and higher T-scores for social isolation (RR 1.07, 95% CI 1.04–1.10) at baseline were each associated with increased risk of all-cause delayed health care access at follow-up in our unadjusted regression. Higher T-scores for emotional support (RR 0.97, 95% CI 0.94–0.99) at baseline were associated with decreased risk of all-cause delayed health care access at follow-up in our unadjusted regression. After adjustment for confounders, higher T-scores for social isolation (aRR 1.05, 95% CI 1.01–1.09) were associated with increased risk of all-cause delayed health care access.

### Health care avoidance attributed to fear of disrespect or mistreatment among SGM parents.

As shown in Table 4, among parents (n = 491), higher scores for identity concealment from health care providers (RR 1.17, 95% CI 1.10–1.25) and higher T-scores for social isolation (RR 1.08, 95% CI 1.04–1.13) at baseline were each associated with increased risk of health care avoidance at follow-up in our unadjusted regression. Higher T-scores for emotional support (RR 0.97, 95% CI 0.94–0.99) at baseline were associated with decreased risk of health care avoidance at follow-up in our unadjusted regression. After adjustment for confounders, higher scores for identity concealment from health care providers (aRR 1.13, 95% CI 1.05–1.22) and higher T-scores for social isolation (aRR 1.06, 95% CI 1.01–1.10) were associated with increased risk of health care avoidance.

## Discussion

In this analysis, we sought to identify social determinants of health that were associated with delayed health care access and health care avoidance in a US-based longitudinal cohort of SGM parents. In our sample of SGM adults, we observed that parents were more likely to report health care avoidance attributed to fear of disrespect or mistreatment compared to non-parents. Among SGM parents, we found that our proxy measures of interpersonal- and community-level social determinants suggested both risk and protective factors that predicted delayed access to necessary health care and health care avoidance attributed to fear of disrespect or mistreatment.

Reports of SGM identity disclosure to health care providers were lower among parents than non-parents. This places SGM parents alongside other SGM subgroups, such as bisexual and ethnoracial minority SGM people, who have been previously shown to demonstrate higher rates of SGM identity concealment from health care providers.^26^ Awareness, or lack thereof, of SGM identity among health care providers may pose a unique challenge given that, compared to SGM non-parents, SGM parents more frequently interface with the health care system in cisheteronormative health care settings (*e.g.*, reproductive health and fertility care clinics, pediatrics care) that thus preclude equitable access to health services.^27^ Prior research has outlined various challenges that SGM parents and their children often face in health care settings, including discrimination, bias, and a lack of recognition of SGM families in the health care system; inappropriate, invasive, or excessive questions from health care providers; and selective SGM identity concealment to avoid compromising the quality of care for their children.^5,6^ Therefore, SGM parents may have had more experiences than SGM non-parents that make them wary of disclosing their SGM identity and/or make them feel that it is unnecessary to do so. We observed that SGM parents and age-matched SGM non-parents were otherwise similar across the remaining demographic characteristics and social determinants of health. Interestingly, we did not find significant differences in annual household income between SGM parents and SGM non-parents, despite the substantial costs associated with family-building for many SGM people.^28^

SGM parents who had higher scores for SGM identity concealment from health care providers were more likely to report health care avoidance attributed to fear of disrespect or mistreatment. When seeking health care for their children, SGM parents, despite being generally willing to disclose their SGM identity to health care providers,^6^ still report numerous barriers related to SGM identity disclosure. Our findings suggest that, when seeking care for themselves, SGM parents may be concerned about many of the barriers previously noted,^29^ perhaps influenced by negative experiences when seeking health care for their children.

When considering community-level social determinants of health among SGM parents, lower social isolation was protective against all-cause delayed health care access and health care avoidance attributed to fear of disrespect or mistreatment. We surmise that participating in interpersonal social, care, and/or support systems likely improves health care access and utilization among SGM parents, especially given evidence that social support among other SGM subgroups mediates improved health outcomes and increased health care utilization.^30^ Community-level support systems may facilitate health care access among SGM people, for example by providing informal referrals to health services, alleviating fears associated with health-seeking behaviors, and/or mitigating challenges with SGM identity disclosure to health care providers.^31^ Other research has noted similar patterns among parents more generally, demonstrating links between poor social support and worse mental health outcomes,^32^ though there is a dearth of research specifically examining the relationship between social support, physical health outcomes, and health care access. Our findings suggest the critical role of community-level support systems as protective factors that facilitate health care access and utilization for SGM parents.

Neither insurance coverage nor having a PCP was associated with all-cause delayed health care access or health care avoidance attributed to fear of disrespect or mistreatment among SGM parents. These findings suggest that delayed health care access among SGM parents may have less to do with passive barriers to health care (*i.e.*, not having insurance coverage or not already having a PCP) and more to do with various behavioral or actionable factors that SGM parents consider when deciding whether to seek care. Drawing upon prior research focused on SGM adults^33^ and SGM parents seeking health care for their children,^5,6^ we surmise that barriers may have a tendency to manifest at the interpersonal level (*e.g.*, identifying SGM-competent health care providers, concerns about how SGM identity disclosure affects the quality of delivered health care).

### Strengths and limitations.

Strengths of this analysis included its application of a social determinants model of health across multiple levels of influence to understand and identify potential targets by which to improve health outcomes and health care access for SGM parents and families. Additionally, this analysis leverages a longitudinal cohort-based study design using data from The PRIDE Study, which uses a community-engaged recruitment, retention, and dissemination strategy and represents a large, diverse, US-based cohort with representation across a wide range of genders, sexual orientations, and geographies.

The majority of participants represented in this analysis self-identified as white, which limits its generalizability to ethnoracial minority parents and their experiences of systemic racism, social determinants of health, and health care access. Given that our data was collected prior to major recent events affecting minoritized communities, including the 2020 COVID-19 pandemic and the 2022 *Dobbs v. Jackson Women’s Health Organization* Supreme Court decision, future analyses are necessary to examine health care access and utilization in a contemporary context. Additionally, developing proactive and detailed data collection mechanisms would allow for further investigation of possible effect modifiers (*e.g.*, family-building strategy, age of children, first-time parent) and a more thorough selection of social determinants of health measures that were not possible given our usage of secondary data.

## Conclusions

Compared to SGM non-parents, SGM parents were less likely to disclose their SGM identity to health care providers and more likely to report health care avoidance due to fear of disrespect or mistreatment. Among our cohort of SGM parents, increased SGM identity concealment from health care providers and higher levels of social isolation were predictive of all-cause delayed health care access and health care avoidance attributed to fear of disrespect or mistreatment. Our analysis underscores the importance of an expansive view of social determinants of health when improving health care access for SGM parents, who likely constitute an SGM subgroup facing unique barriers. Approaches focused on interpersonal- and community-level factors, such as building and strengthening support systems among SGM parents and families, may be combined with other systemic and structural approaches that promote health equity.^34^ Given the increasing rate at which SGM people are growing their families against the background of worsening SGM acceptance,^35^ developing supportive mechanisms for SGM parents and families is timely and will improve the health and well-being of the growing population of SGM parents and their children.

## Supplementary Material

Supplementary Files

This is a list of supplementary files associated with this preprint. Click to download.

• Appendix.docx

## Figures and Tables

**Table 1:** Demographic characteristics of SGM parents and non-parents at baseline, The PRIDE Study, 2018

		Parents	Non-parents
	**(n)**	555	555
**Age**	**(mean, IQR)**	44 (37–56)	44 (37–56)
**Race and ethnicity** *	**(n, %)**		
American Indian or Alaska Native		20 (3.6)	27 (4.9)
Asian		16 (2.9)	21 (3.8)
Black, African American, or African		16 (2.9)	12 (2.2)
Hispanic or Latinx		26 (4.7)	45 (8.1)
Middle Eastern or North African		6(1.1)	5 (0.9)
Native Hawaiian or Pacific Islander		2 (0.4)	3 (0.5)
White		523 (94.2)	514 (92.6)
Another race or ethnicity not listed		19 (3.4)	4 (0.7)
Selected multiple		38 (6.8)	36 (6.5)
**Gender groups**	**(n, %)**		
Cisgender man		127 (22.9)	264 (47.6)
Cisgender woman		238 (42.9)	145 (26.1)
Gender diverse AFAB**		69 (12.4)	63 (11.4)
Gender diverse AMAB***		10 (1.8)	19 (3.4)
Transgender man		44 (7.9)	35 (6.3)
Transgender woman		57 (10.3)	23 (4.1)
Sexual orientation*	**(n, %)**		
Asexual		30 (5.4)	35 (6.3)
Bisexual		179 (32.3)	92 (16.6)
Gay		135 (24.3)	295 (53.2)
Lesbian		171 (30.8)	113 (20.4)
Pansexual		97 (17.5)	52 (9.4)
Queer		171 (30.8)	161 (29.0)
Questioning		16 (2.9)	6 (1.1)
Same-gender loving		29 (5.2)	19 (3.4)
Straight		23 (4.1)	9 (1.6)
Another orientation not listed		19 (3.4)	10 (1.8)
Selected multiple		191 (34.4)	160 (28.8)
**Annual household income**	**(n, %)**		
$0 – $20,000		48 (8.6)	67 (12.1)
$20,001 – $50,000		83 (15.0)	113 (20.4)
$50,001 – $80,000		108 (19.5)	98 (17.7)
$80,001 – $100,000		76 (13.7)	66 (11.9)
$100,001 – $150,000		230 (41.4)	203 (36.6)
Missing		10 (1.8)	8 (1.4)
**Marital status**	**(n, %)**		
Married, civil union, or domestic partnership		350 (63.1)	199 (35.9)
Never in legally recognized partnership		39 (7.0)	299 (53.9)
Widowed, divorced, or separated		161 (29.0)	57 (10.3)
Missing		5 (0.9)	0 (0.0)
**In a relationship**	**(n, %)**	457 (82.6)	342 (61.6)
**Reported having a disability**	**(n, %)**	139 (25.1)	126 (22.7)
**Insurance status and type**	**(n, %)**		
Uninsured		8 (1.4)	21 (3.8)
Private		392 (70.6)	393 (70.8)
Public		97 (17.5)	101 (18.2)
VA/TRICARE		19 (3.4)	23 (4.1)
Other		6(1.1)	7 (1.3)
Missing		33 (5.9)	10 (1.8)
**Has a primary care provider**	**(n, %)**	480 (89.6)	487 (88.5)
**PROMIS measures**	**(mean, SD)**		
Emotional support T-scores		52.25 (8.12)	52.79 (8.46)
Social isolation T-scores		54.95 (7.16)	54.42 (7.25)
**Modified Nebraska Outness Scale**	**(mean, SD)**		
Identity disclosure to health care providers		7.10 (3.56)	7.89 (3.07)
Identity concealment from health care providers		1.66 (2.75)	1.56 (2.64)

* Participants could select more than one response; therefore, percentages may sum to greater than 100%

** Assigned female at birth

*** Assigned male at birth

**Table 2:** Healthcare utilization among SGM parents and non-parents at follow-up, The PRIDE Study, 2019

		Parents	Non parents	value
	**(n)**	555	555	
**Avoided health care due to fear of being disrespected ormistreated in the past 12 months**	**(n,%)**	68 (13.8)	46 (9.0)	0.02
**Delayed necessary health care in the past 12 months**	**(n,%)**	98 (20.1)	91 (18.0)	0.45
**Did not receive necessary health care in the past 12months**	**(n,%)**	64 (13.1)	55 (10.9)	0.34
**Reasons for delayed care** *	**(n,%)**			
My insurance company wouldn’t approve, cover, or pay for care		37 (40.7)	41 (41.8)	0.99
The health care provider could not schedule me in a timely fashion		28 (30.8)	25 (25.5)	0.52
I couldn’t afford care		26 (28.6)	34 (34.7)	0.46
Another reason not listed		15 (16.5)	20 (20.4)	0.61
Problems getting to health care provider's office		8 (8.8)	9 (9.2)	1.00
I couldn’t get time off work or school		8 (8.8)	10 (10.2)	0.93
I thought I would be mistreated or disrespected on the basis of my gender identity		8 (8.8)	14 (14.3)	0.34
I didn’t have time or took too long		8 (8.8)	13 (13.3)	0.46
Health care provider refused to accept the insurance plan		7 (7.7)	9 (9.2)	0.92
I don’t know where to go to get care		6 (6.6)	5 (5.1)	0.90
I was refused services		5 (5.5)	3 (3.1)	0.64
I thought I would be mistreated or disrespected on the basis of my sexual orientation		3 (3.3)	3 (3.1)	1.00
I couldn’t get child care		2 (2.0)	0 (0.0)	0.51

* Percentages are among individuals who reported delayednecessary health care in the past 12 months

**Table 3:** Cohort analysis of social determinants associated with delayed health care among SGM parents (N = 488), The PRIDE Study, 2018 to 2019

	Unadjusted		Adjusted*	
	RR (95% CI)	value	aRR (95% CI)	value
**Community-level**				
Emotional support T-scores	0.97 (0.94, 0.99)	0.004	0.98 (0.95, 1.00)	0.09
Social isolation T-scores	1.07 (1.04, 1.1)	<0.001	1.05 (1.01, 1.09)	0.009
**Interpersonal-level**				
Identity concealment from health care providers	1.09 (1.03, 1.16)	0.005	1.04 (0.97, 1.12)	0.22
Has a primary care provider	0.93 (0.65, 1.34)	0.71	0.96 (0.86, 1.09)	0.56
**Individual-level**				
Insurance status and type				
Private	ref		ref	
Uninsured	0.78 (0.11, 5.6)	0.80	1.13 (0.14, 8.89)	0.91
Public	1.34 (0.82, 2.2)	0.24	1.11 (0.59, 2.11)	0.75
VA/TRICARE	1.36 (0.5, 3.73)	0.55	1.51 (0.52, 4.35)	0.45
Other	0.91 (0.13, 6.53)	0.92	1. 34 (0.15, 11.98)	0.80

67 (12.1%) of parents were missing data on delayed health care

* Adjusted for gender group, age, household income, and disability status

**Table 4:** Cohort analysis of social determinants associated with health care avoidance attributed to fear of disrespect or mistreatment among SGM parents (N = 491), The PRIDE Study, 2018 to 2019

	Unadjusted		Adjusted*	
	RR (95% CI)	value	aRR (95% CI)	value
**Community-level**				
Emotional support T-scores	0.97 (0.94, 0.99)	0.02	0.98 (0.95, 1.01)	0.22
Social isolation T-scores	1.08 (1.04, 1.13)	<0.001	1.06 (1.01, 1.10)	0.01
**Interpersonal-level**				
Identity concealment from health care providers	1.17 (1.1, 1.25)	<0.001	1.13 (1.05, 1.22)	0.002
Has a primary care provider	0.59 (0.3, 1.15)	0.12	0.75 (0.37, 1.54)	0.43
**Individual-level**				
Insurance status and type				
Private	ref		ref	
Uninsured	NA		NA	
Public	1.75 (0.99, 3.09)	0.05	2.03 (0.96, 4.29)	0.07
VA/TRICARE	1.94 (0.7, 5.43)	0.21	2.46 (0.84, 7.22)	0.10
Other	NA		NA	

64 (11.5%) of parents were missing data on health care avoidance

* Adjusted for gender group, age, household income, and disability status

## Data Availability

The datasets generated and/or analysed during the current study are not publicly available due to participant privacy but are available from the corresponding author on reasonable request. We welcome the opportunity to facilitate high-quality, community-engaged research collaborations that aim to improve the health and wellbeing of LGBTQIA+ communities. Through The PRIDE Study’s ancillary studies, a wide variety of investigators working on academic or community-based projects related to LGBTQIA+ health can apply to work collaboratively with The PRIDE Study team and access data. For more information, please visit: https://pridestudy.org/collaborate.
